# Cultural adaptation and validation for Brazilian portuguese of the MENTOR tool: monitoring the efficacy of neurogenic bowel treatment

**DOI:** 10.1038/s41393-026-01203-3

**Published:** 2026-04-24

**Authors:** Natália MB Bezerra, Lilian L. Lisboa, Romulo AL Vasconcelos, Klaus Krogh, Cristiano M. Gomes, Adriano A. Calado

**Affiliations:** 1 Universidade de Pernambuco, Recife, PE Brazil; 2 Instituto Santos Dumont, Macaíba, RN Brazil; 3 Department of Hepatology and Gastroenterology, Aarhus University Hospital, Aarhus, Denmark; 4 Universidade de São Paulo, São Paulo, SP Brazil

**Keywords:** Neurological disorders, Diagnosis, Digestive signs and symptoms

## Abstract

**Study design:**

Cross-sectional study of cultural adaptation and validation.

**Objectives:**

To translate and validate the MENTOR Tool, an instrument designed to monitor the effectiveness of treatment for neurogenic bowel dysfunction (NBD) and to support more objective clinical decision-making in individuals with spinal cord injury (SCI).

**Setting:**

Individuals with SCI treated at the Anita Garibaldi Health Education and Research Center, Macaiba/RN, and at Oswaldo Cruz University Hospital, Recife/PE, Brazil.

**Methods:**

Cross-cultural adaptation was conducted at two specialized referral centers for SCI, following the international methodology proposed by Guillemin et al. The MENTOR Tool was administered as an interview and reapplied after an interval of 7 to 21 days. In addition, participants completed the Gastrointestinal Symptom Rating Scale (GSRS). The following measurement properties were evaluated: reproducibility, internal consistency, reliability, and construct validity.

**Results:**

50 individuals participated in the study. The MENTOR Tool demonstrated excellent reproducibility, with an intraclass correlation coeficiente (ICC) of 0.974, Internal consistency was acceptable, with a Cronbach’s alpha of 0.61. Construct Validity was supported by significant correlations between the final Neurogenic Bowel Dysfunction Score (NBDS) and GSRS scores, as well as between GSRS scores and the final MENTOR rating. Additionally, statistically significant differences were observed among the three MENTOR categories.

**Conclusions:**

The MENTOR tool was successfully translated and validated into Brazilian Portuguese, demonstrating reliability and reproducibility for monitoring bowel treatment effectiveness in individuals with SCI and supporting clinical decision-making in the management of NBD.

## Introduction

In Brazil, there are around 6.000 to 8.000 new cases of spinal cord injury (SCI) every year. 80% of the victims are men and 60% are between the ages of 10 and 30, representing a high incidence when compared to other countries [1, 2].

SCI can lead to incoordination of the nervous system in sensory and motor control, which can generate significant chronic disabilities, as well as vesico-sphincter dysfunctions, with repercussions on the individual’s functionality. Failure of voiding and defecation reflexes in individuals with spinal cord injury can lead to neurogenic bladder and bowel dysfunction, in which the inability to control voiding and defecation negatively impacts autonomy and social participation. [3, 4].

Neurogenic bowel dysfunction (NBD) has clinical features such as constipation and fecal incontinence, as well as pain, discomfort and abdominal distension. It is the cause of hospitalizations for complications such as fecal impaction, megacolon and volvulus, which occur twice as often in individuals with neurological disease [5, 6].

The general aim of NBD management is to maintain regular rectal emptying while preventing fecaloma formation and ensuring fecal continence. However, current guidelines provide limited detail on intestinal alterations, making it difficult for clinicians to establish an accurate diagnosis and develop appropriate therapies. This limitation often leads to treatment failure, with high rates of inadequate bowel function control and patient dissatisfaction [4, 5].

In Brazil, to date, there is no standardized instrument or recommendation to evaluate the effect of treatment strategies on NBD symptoms in adults with SCI.

Recently, a Brazilian Portuguese translation, cultural adaptation, and semantic validation of the pediatric Neurogenic Bowel Dysfunction Score (NBDS) was published, providing an important tool for children and adolescents with NBD in Brazil [7]. However, there is still no validated multidimensional clinical decision support tool for adults with spinal cord injury and NBD that combines a symptom-based severity score, patient-reported satisfaction, and ‘red flag’ symptoms to guide therapeutic adjustments. Our study addresses this gap by translating and validating the MENTOR tool for Brazilian Portuguese in an adult SCI population.

The MENTOR (Monitoring Efficacy of Neurogenic Bowel Treatment On Response) is a clinical decision support tool designed to monitor the effectiveness of treatment for neurogenic bowel dysfunction. The tool is based on a questionnaire comprising three dimensions: objective measures derived from the Neurogenic Bowel Dysfunction Score (NBDS) [8], which quantify the severity of neurogenic bowel dysfunction; the patient’s subjective perception of bowel function (good, adequate, bad, or very bad); and bowel symptoms requiring special attention. By integrating information from the NBDS score, patient-reported satisfaction, and symptoms of special attention, the MENTOR matrix classifies each individual within a traffic-light system. In this system, the green zone (“Monitor”) indicates adequate bowel management; the yellow zone (“Discuss”) reflects suboptimal treatment requiring discussion with the patient, which may or may not result in further investigation or modification of treatment; and the red zone (“Act”) represents inadequate treatment, for which further investigation and a change in treatment are required [9].

This tool was tested against the blinded clinical judgment of international gastroenterology and rehabilitation specialists from the UK, Denmark, the USA, Italy, the Netherlands, and Germany, demonstrating good agreement between MENTOR recommendations and expert opinion [9]. In addition, the questionnaire has already been translated into Dutch, Finnish, French, German, Italian, and Spanish [10].

Given the absence of a validated clinical decision support tool for NBD in Brazilian adults with SCI, the aim of this study was to translate the MENTOR tool into Brazilian Portuguese and evaluate its measurement.

## Methods

This cross-sectional validation study was conducted at two specialized centers: the Anita Garibaldi Health Education and Research Center (Macaíba/RN) and the Oswaldo Cruz University Hospital (Recife/PE), both as referral centers for bladder and bowel dysfunction after SCI in their respective states of Brazil.

### Ethics approval and consent to participate

The study was approved by the Research Ethics Committee of the HUOC/PROCAPE Hospital Complex (CAAE 57861422.7.0000.5192), and all methods were performed in accordance with the relevant guidelines and regulations, including Resolution 466/2012 of the National Health Council and its complementary resolutions. Informed consent was obtained from all participants.

### Population and sample

The population of this study was made up of individuals diagnosed with traumatic spinal cord injury who presented for a routine medical visit were invited to participate. The sample was made up of 50 participants of both sexes aged 18 years or older. Participants with cognitive impairment of any degree, inflammatory bowel disease even without activity, patients with a history of gastroenterological malignancy and intestinal stoma were not eligible for the study. Only one participant missed the second interview and was therefore excluded from the final sample (n = 50).

Participants were consecutively invited during routine outpatient visits at the two centers, which, in addition to providing outpatient medical care, also offer motor rehabilitation and other services. Consequently, participants attend the centers on a weekly basis, which facilitated data collection. After consent, the interview was conducted during the same clinical visit, and a second interview was scheduled 7–21 days later, preferably during a subsequent planned visit or rehabilitation session. No financial incentive was provided; adherence was facilitated by aligning research assessments with usual care appointments and by reminder contacts.

MENTOR results were available to clinicians; however, no systematic protocol for treatment modification based on the tool was implemented during the study. We acknowledge that unstructured changes in management could potentially influence retest scores and discuss this as a limitation.

### Translation and cultural adaptation

After authorization from the author of the questionnaire, Klaus Krogh, the translation and validation process began.

**Fig. 1:**
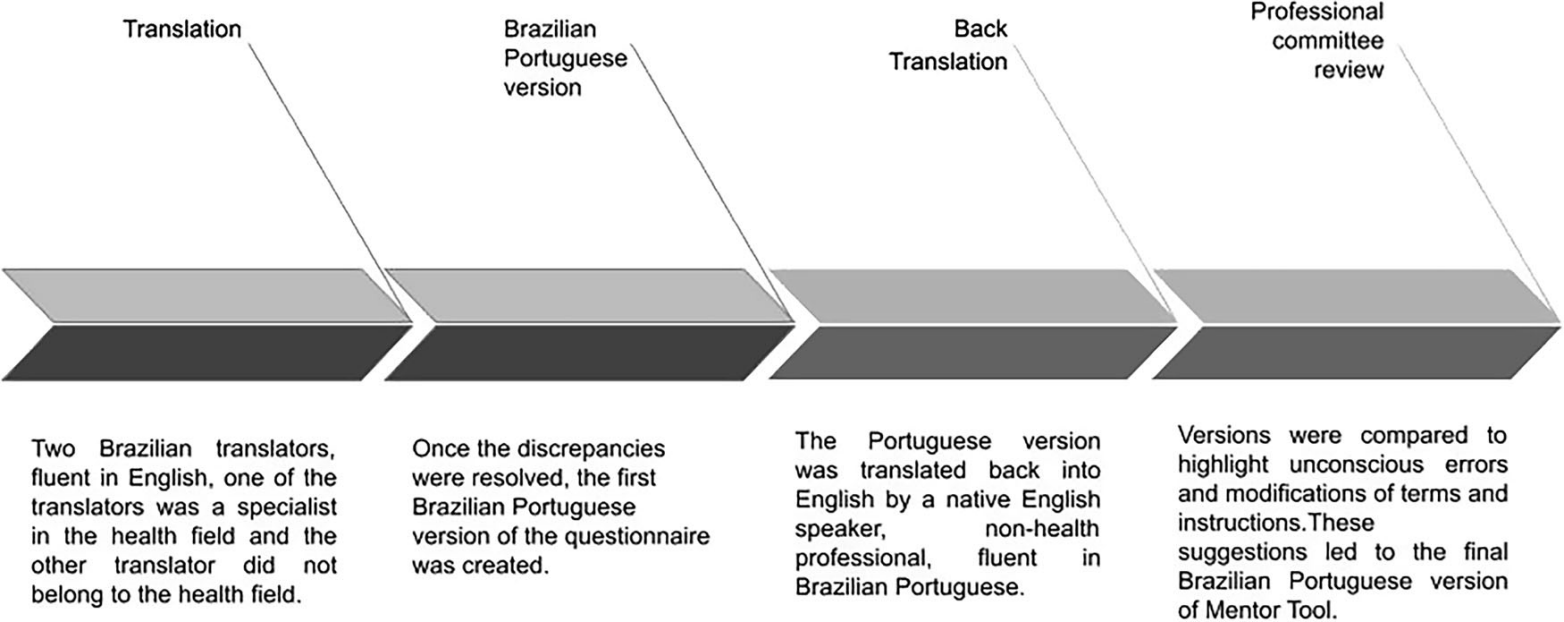
Flowchart of the translation process and the Portuguese version of the Mentor Tool. Four-step process: (1) forward translation by two Brazilian bilingual translators (one with and one without a health background); (2) synthesis and development of the first Brazilian Portuguese version; (3) back-translation by a native English speaker fluent in Brazilian Portuguese; and (4) expert committee review, including comparison of versions and resolution of discrepancies, resulting in the final version. Arrows indicate the sequence of steps.

The translation and cultural adaptation process followed the international methodology proposed by F. Guillemin (1.995) [11]. The translation process was conducted by two Brazilian translators fluent in English: one physiotherapist and one translator without a health background. Both provided written reports highlighting translation choices and terms requiring special attention. Discrepancies were resolved through discussion and consensus. The consolidated Portuguese version was then back-translated into English by a native English speaker fluent in Portuguese, with no health background. The back-translation was compared with the original English version by the research team to identify potential semantic or conceptual inconsistencies. Finally, the translated version was reviewed by a multidisciplinary committee of bilingual health professionals, who reached consensus on the final Brazilian Portuguese version of the MENTOR Tool Fig. 1.

### Validation

The test version of the MENTOR tool was administered in the form of an interview by the same evaluator to assess comprehension and applicability of the questions. To evaluate reliability, the MENTOR tool was reapplied after an interval of 7 to 21 days. Test–retest reliability was assessed using the intraclass correlation coefficient (ICC) for continuous scores and Cohen’s kappa for categorical classification.

At the second interview, in addition to the participants answering the test version of the MENTOR Tool, the Gastrointestinal Symptom Rating Scale (GSRS) questionnaire [12], which served as the comparator patient-reported outcome measure to assess construct validity through correlations between domains of the two instruments.

The GSRS is a validated generic gastrointestinal symptom questionnaire available in Brazilian Portuguese. However, it is not specific to neurogenic bowel dysfunction and was not designed to provide a clinical decision-making algorithm for adjusting bowel management in individuals with SCI [12].

### Data analysis

Descriptive statistics were used to characterize the sample. Absolute (n) and relative (%) frequencies were calculated for categorical variables, while means and medians were reported for continuous variables. Descriptive statistics for the MENTOR and GSRS instruments are presented in Supplementary Material, Tables 1 and 2.

To assess test-retest reproducibility, the intraclass correlation coefficient (ICC) and the Kappa agreement measure between the MENTOR questionnaire values at the two analyzed moments were used. ICC values of less than 0.5 indicate low reliability, values between 0.5 and 0.75 indicate moderate reliability, values between 0.75 and 0.9 indicate good reliability and values greater than 0.90 indicate excellent reliability [13].

Internal consistency and reliability were assessed by Cronbach’s alpha, with the following thresholds: Very low < 0.30; Low 0.30 - 0.59; Moderate 0.60 - 0.74; High 0.75 - 0.89; Very high > 0.90 [14].

Construct validity was examined by comparing MENTOR tool domains with the Gastrointestinal Symptom Rating Scale (GSRS) using Pearson’s correlation coefficients. Additionally, analysis of variance (ANOVA) was applied to compare mean scores across relevant groups.

All analyses were performed using IBM SPSS Statistics version 21.0 (IBM Corp., Armonk, NY, USA). A two-sided significance level of 5% was adopted.

### Sample size calculation

Sample size considerations followed Consensus-based Standards for the selection of health Measurement Instruments (COSMIN) recommendations for validation studies of patient-reported outcome measures, which suggest that samples of at least 50 participants are adequate for reliability and construct validity analyses [15, 16]. Assuming an expected ICC ≈ 0.80, n = 50 yields a 95% confidence interval half-width of ~0.15, and provides ~80% power (α = 0.05) to detect a correlation of r ≈ 0.40 with the GSRS. Accounting for possible attrition between test and retest, we targeted 50 participants.

## Results

Fifty individuals took part in this study, 39 (78%) men and 11 (22%) women, with an average age of 39.2 ± 10.9 years. With regard to schooling, 66% of the individuals had between 5 and 13 years of schooling, 4% had not studied, 24% had up to 3 years and 6% had graduated from college. Only one participant was excluded because he failed to reapply the questionnaire.

The level of injury of the individuals interviewed was described based on clinical records and categorized anatomically as upper cervical (4.1%), lower cervical (10.2%), upper thoracic (23.53%), middle thoracic (24.5%), and lower thoracic (34.7%).

The translation and back-translation process showed no disagreement and the existing considerations did not change the semantics so that the reader could achieve complete understanding.

The MENTOR Tool showed an intraclass correlation (ICC) of 0.974, indicating excellent reproducibility. Most of the questions had excellent values, with the exception of questions Q3 (Do you experience uneasiness, sweating or headaches during or after defaecation?) and Q9 (Do you experience uncontrollable flatus?) which had ICCs below 0.8, as shown in Table 1. It was not possible to calculate the ICC for item Q8 (Do you take medication to treat faecal incontinence?), as all the participants answered “no” in both the first assessment and the retest, since the sample did not use such medication.

**Table 1:** Results of the MENTOR Tool reproducibility analysis - Intraclass Correlation Coefficient (ICC) and Kappa measure between the two moments analyzed.

VARIABLES	ICC (IC95%)	KAPPA (p-value)
Q1 - How often do you defaecate	0.921 (0.860 – 0.955)	0.851 (p < 0.001)
Q2 - How much time do you spend on each defaecation	0.911 (0.843 – 0.950)	0.802 (p < 0.001)
Q3 - Do you experience uneasiness, sweating or headaches during or after defaecation	0.791 (0.630 – 0.882)	0.649 (p < 0.001)
Q4 - Do you take medication (tablets) to treat constipation	1.0 (1.0 – 1.0)	1.0 (p < 0.001)
Q5 - Do you take medication (drops or liquid) to treat constipation	1.0 (1.0 – 1.0)	1.0 (p < 0.001)
Q6 - How often do you use digital evacuation	0.936 (0.886 – 0.964)	0.878 (p < 0.001)
Q7 - How often do you have involuntary defaecation	0.943 (0.898 – 0.968)	0.863 (p < 0.001)
Q8 - Do you take medication to treat faecal incontinence	-	-
Q9 - Do you experience uncontrollable flatus	0.778 (0.594 – 0.877)	0.632 (p < 0.001)
Q10 - Do you have peri-anal skin problems	1.0 (1.0 – 1.0)	1.0 (p < 0.001)
NBDS score	0.926 (0.870 – 0.958)	0.718 (p < 0.001)
General Satisfaction with your bowel management	0.852 (0.738 – 0.916)	0.262 (p < 0.001)
Satisfaction with your bowel functions over the past 4 weeks	0.944 (0.900 – 0.968)	0.868 (p < 0.001)
Classification of the MENTOR tool	0.974 (0.954 – 0.985)	0.840 (p < 0.001)

*NBDS* neurogenic bowel dysfunction score.

Items are presented here in English for readability; the validate questionnaire was administered in Brazil Portuguese

The internal consistency of the MENTOR tool, assessed using Cronbach’s alpha, was 0.61, indicating moderate reliability. No negative correlations were observed among the items, and for those items with variability, the removal of any item reduced the overall alpha, confirming that each contributes meaningfully to the construct being measured. Item Q8 could not be evaluated because all participants answered “no,” resulting in no variance for this item.

The construct validity was significant, as there was a correlation between the final NBDS score and the General GSRS. When we look at the domains separately, only the Reflux domain and the Indigestion domain showed no statistically significant correlation with the NBDS score Table 2.

**Table 2:** Result of the validity of the convergent construct of the NBDS score with the GSRS.

GSRS	C.Correlation (p-value)
GSRS General	0.454 (p < 0.001)*
GSRS Abdominal Pain	0.609 (p < 0.001)*
GSRS Reflux	0.069 (p = 0.635)
GSRS Indigestion	0.187 (p = 0.193)
GSRS Diarrhea	0.406 (p = 0.003)*
GSRS Constipation	0.403 (p = 0.004)*

^*^ p < 0.05 (statistical significant)

*NBDS* neurogenic bowel dysfunction score, GSRS gastrointestinal symptom rating scale

When we compared the GSRS values with the final MENTOR Tool score, we found a statistically significant difference in the means between the three MENTOR scores. The “Act” (red zone) group showed higher GSRS values than the “Monitor” (green zone) and “Discuss” (yellow zone) groups, with the exception of the Reflux and Indigestion domains, where there were no relevant differences between the MENTOR Tool classes. Table 3

**Table 3:** Comparison of GSRS values according to MENTOR score - Mean (SD).

GSRS	MENTOR score			p-value
	Monitor (n = 25)	Discuss (n = 9)	Act (n = 16)	
GSRS General	29.7 (8.5)	30.0 (5.8)	41.1 (9.8)	< 0.001*
Abdominal Pain	4.3 (1.6)	3.8 (0.9)	6.6 (1.0)	< 0.001*
Reflux	3.0 (1.9)	3.9 (2.4)	3.6 (1.3)	0.409
Indigestion	11.4 (5.2)	13.4 (4.8)	13.9 (3.6)	0.225
Diarrhea	4.0 (1.5)	3.7 (1.0)	7.1 (4.1)	0.001*
Constipation	6.9 (2.6)	5.2 (1.1)	10.0 (2.9)	< 0.001*

^*^ p < 0.05 (statistical significant)

*GSRS* gastrointestinal symptom rating scale

## Discussion

In the Brazilian Portuguese translation, the MENTOR Tool demonstrated excellent test–retest reliability (ICC = 0.974), with particularly high agreement in the “Act” (red zone) category, in which 100% agreement was observed. Importantly, this finding reflects the stability of classification over a short retest interval (7–21 days) rather than a lack of clinical improvement. No therapeutic intervention was implemented between assessments, as this could have interfered with the psychometric validation of the instrument. Moreover, individuals classified in the “Act” category represent patients with more severe and chronic neurogenic bowel dysfunction, in whom meaningful clinical changes are less likely to occur over a short period without targeted intervention. Similar patterns of higher agreement in the most severe categories were also reported in the original validation study [8], supporting the robustness of the tool in identifying patients requiring active management.

Considering that Cronbach’s alpha is influenced by the dimensionality of an instrument and that multidimensional scales tend to present lower alpha values, the observed Cronbach’s alpha of 0.61 indicates acceptable internal consistency when interpreted in context [17]. Although a value of 0.70 is traditionally cited as a reference, the COSMIN guidelines emphasize that reliability estimates should be interpreted considering the construct being measured and the purpose of the instrument, particularly in patient-reported outcome measures and during cross-cultural validation [16].

Item Q8 (“Do you take medication to treat faecal incontinence?”) could not be evaluated because it showed no variability, as all participants answered “no” in both interviews. Consequently, it was not possible to formally assess its reliability or its contribution to internal consistency. The uniform negative responses likely reflect the low use of medication to prevent faecal incontinence in this sample, rather than providing direct evidence of item comprehension. This finding is consistent with the clinical profile of individuals with SCI, in whom stool loss and diarrhea are generally less prevalent than constipation, and bowel management strategies primarily aim to stimulate defecation and prevent unexpected stool loss [18]. Accordingly, individuals tend to use constipation-related medications, such as laxatives and enemas, more frequently than medications specifically intended to prevent faecal incontinence [19].

When comparing the GSRS to the NBDS, the overall score and the domains abdominal pain, diarrhea and constipation have significant and high correlations with each other, measuring similar aspects of gastrointestinal symptom burden associated with NBD. The reflux and indigestion domains showed no correlation, as there is no item that assesses these functions in the translated tool.

In our sample, the strongest correlation between the NBDS and GSRS domains was observed for abdominal pain, whereas correlations with constipation and diarrhea were moderate. This pattern may be associated with the high frequency of abdominal discomfort reported by Brazilian adults with SCI and NBD, as well as with the content of the NBDS, which includes items related to bowel evacuation characteristics and associated discomfort that may overlap with the perception of abdominal pain. The significantcorrelations with the constipation and diarrhea domains are consistent with the symptom profile typically observed in NBD.

There was a statistically significant difference in mean MENTOR final ratings when compared with GSRS scores, with a good correlation between the instruments. Participants classified in the “Act” group (red) presented higher GSRS scores than those in the “Monitor” (green) and “Discuss” (yellow) groups, indicating more severe gastrointestinal symptoms and a potential need for treatment review. Although GSRS scores were significantly higher in the Act group, differences between the Monitor and Discuss categories were modest for some domains. This pattern is consistent with the design of MENTOR as a clinical decision support tool: the Discuss category (yellow) represents patients with suboptimal but not clearly failing management, for whom decisions rely on shared decision-making rather than symptom scores alone. Accordingly, weaker differentiation between Monitor (green) and Discuss (yellow) on the GSRS does not undermine the utility of MENTOR but rather reflects its intended nuanced guidance, whereas the GSRS alone does not integrate NBD-specific severity, patient satisfaction, or red-flag symptoms into a comprehensive decision-making framework. [9, 12].

For the academic community, the availability of a validated Brazilian Portuguese version of the MENTOR tool enables standardized, evidence-based assessment, facilitates consistent data collection across institutions, supports multicenter collaboration, and allows Brazilian cohorts to be compared with international populations.

This study had some limitations that should be taken into account when interpreting the results. The sample size was relatively small, although compatible with cross-cultural validation studies, which may limit the generalizability of the findings. In addition, the restriction to patients with traumatic spinal cord injury from two specialized centers which may reflect specific clinical characteristics of these services which may limit generalizability to other regions or neurological populations, such as multiple sclerosis or spina bifida. None of the participants used medication to prevent stool loss, which made it impossible to analyze the reproducibility of one of the instrument’s items. Future studies with larger and more heterogeneous samples could confirm and expand on these findings.

## Conclusion

The MENTOR tool was translated and validated into Brazilian Portuguese with acceptable reliability and excellent reproducibility to monitor the efficacy of bowel treatment in individuals with traumatic spinal cord injury and to assist in clinical decision-making on the management of neurogenic bowel dysfunctions and strengthen participation in global collaborative studies.

## Supplementary information

Descriptive Data of the Study Sample

Portuguese version of the Mentor Tool

## Data Availability

The data sets generated and analyzed during the current study are available from the corresponding author upon reasonable request.
