# Supplementary material for: Cultural adaptation and validation for Brazilian portuguese of the MENTOR tool: monitoring the efficacy of neurogenic bowel treatment
Source: Spinal Cord. 2026 Apr 24;64(7):618–22. doi: 10.1038/s41393-026-01203-3 (PMC13345958; doi:10.1038/s41393-026-01203-3)
Supplement: Supplementary file 1 — Descriptive Data of the Study Sample [file 41393_2026_1203_MOESM1_ESM.docx]

**Supplementary Material**

**Table 1.** Descriptive profile of the study population based on NBDS scores and MENTOR classification (N = 50)

| **Variables** | **n( %) or Mean (SD)/ Median (range)** |
| --- | --- |
| **NBDS total score**  Mean (SD)  Median | 10.6 (7.8)  9 (1-34) |
| **NBDS Classification** |  |
| Very mild (0-6) | 19 (38%) |
| Mild (7-9) | 9 (18%) |
| Moderate (10-13) | 7 (14%) |
| Severe (14+)  **Overall satisfaction**  Mean (SD)  Median (min-max)  **Satisfaction with bowel function over**  Good  Adequate/acceptable  Poor  Very poor  **Symptoms since last consultation**  None  Some symptoms  **MENTOR classification**  Monitor  Discuss  Act | 15 (30%)  5.6 (2.7)  6 (0-10)  17 (34%)  19 (38%)  12 (24%)  2 (4%)  31 (62%)  19 (38%)  25 (50%)  9 (18%)  16 (32%) |

NBDS - Neurogenic Bowel Dysfunction Score

**Table 2.** Descriptive statistics for GSRS total score and domain scores (N=50)

| **Variables** |  | **Mean** | **SD** | **Median** | **Min-Max** |
| --- | --- | --- | --- | --- | --- |
| **GSRS total** |  | 33.4 | 9.9 | 32.0 | 18-58 |
| **Abdominal pain** |  | 4.9 | 1.8 | 5.0 | 3-9 |
| **Reflux** |  | 3.3 | 1.9 | 2.0 | 2-9 |
| **Indigestion** |  | 12.6 | 4.8 | 12.0 | 4-22 |
| **Diarrhea** |  | 5.0 | 2.9 | 4.0 | 3-14 |
| **Constipation** |  | 7.6 | 3.0 | 6.0 | 3-15 |

GSRS total score was calculated as the sum of all items (Q1–Q15). Domain scores were calculated as follows: abdominal pain (Q1, Q4, Q5), reflux (Q2, Q3), indigestion (Q6–Q9), diarrhea (Q11, Q12, Q14), and constipation (Q10, Q13, Q15)..
