# Supplementary material for: Cultural adaptation and validation for Brazilian portuguese of the MENTOR tool: monitoring the efficacy of neurogenic bowel treatment
Source: Spinal Cord. 2026 Apr 24;64(7):618–22. doi: 10.1038/s41393-026-01203-3 (PMC13345958; doi:10.1038/s41393-026-01203-3)
Supplement: Supplementary file 2 — Portuguese version of the Mentor Tool [file 41393_2026_1203_MOESM2_ESM.pdf]

Ferramenta MENTOR

Nome do paciente:
Data da coleta:

Seção 1: **Escala de Disfunção do Intestino Neurogênico (Neurogenic Bowel Dysfunction Score - NBDS)**

Marque apenas uma resposta por pergunta. Suas respostas devem refletir sua situação atual.

**1**	**Com que frequência você evacua?**		**Pontos**
☐	Diariamente	(0 pontos)	
☐	1 a 6 vezes por semana	(1 ponto)	
☐	Menos de uma vez por semana	(6 pontos)	
**2**	**Quanto tempo você demora em cada evacuação?**		**Pontos**
☐	Menos de 30 minutos	(0 pontos)	
☐	31 a 60 minutos	(1 ponto)	
☐	Mais de uma hora	(7 pontos)	
**3**	**Você sente desconforto, tem suor ou dores de cabeça durante ou após a evacuação?**		**Pontos**
☐	Sim	(2 pontos)	
☐	Não	(0 pontos)	
**4**	**Você toma medicamentos (comprimidos) para tratar o intestino preso?**		**Pontos**
☐	Sim	(2 pontos)	
☐	Não	(0 pontos)	
**5**	**Você toma medicamentos (gotas ou líquido) para tratar o intestino preso?**		**Pontos**
☐	Sim	(2 pontos)	
☐	Não	(0 pontos)	
**6**	**Quantas vezes você usa os dedos para ajudar na evacuação?**		**Pontos**
☐	Menos de uma vez por semana	(0 pontos)	
☐	Uma vez ou mais por semana	(6 pontos)	
**7**	**Com que frequência você tem perda de fezes sem controle?**		**Pontos**
☐	Todo dia	(13 pontos)	
☐	1 a 6 vezes por semana	(7 pontos)	
☐	3 a 4 vezes por mês	(6 pontos)	
☐	Algumas vezes por ano ou menos	(0 ponto)	
**8**	**Você toma medicamentos para evitar a perda de fezes?**		**Pontos**
☐	Sim	(4 pontos)	
☐	Não	(0 pontos)	
**9**	**Você deixa escapar gazes sem controle?**		**Pontos**
☐	Sim	(2 pontos)	
☐	Não	(0 pontos)	
**10**	**Você tem problemas na pele ao redor do ânus?**		**Pontos**
☐	Sim	(3 pontos)	
☐	Não	(0 pontos)	

**Interpretação da Escala**	
**Pontuação 0-6 : Muito leve**	**Pontuação 7-9 : Leve**
**Pontuação 10-13 : Moderada**	**Pontuação 14+: Severa**

Seção 2: **Satisfação subjetiva do paciente**

Pergunte ao seu paciente:

Como você avaliaria a sua satisfação* com suas funções intestinais ao longo das 4 últimas semanas?

*Sensação subjetiva de satisfação em geral

- ☐ Boa
- ☐ Adequada/aceitável
- ☐ Ruim
- ☐ Muito ruim

Seção 3: **Sintomas que requerem atenção especial**

Pergunte ao seu paciente:

Você apresentou algum destes sintomas desde a sua última consulta médica referente a problemas intestinais?

Marque todos os casos que se aplicam. Se nenhum se aplicar, marque “*nenhum dos casos anteriores*”.

- ☐ Dor intensa no abdômen ou no reto.
- ☐ Novo ou aumento de sangramento pelo ânus
- ☐ Hospitalização decorrente de problemas intestinais
- ☐ Redução da sua independência com relação aos cuidados intestinais
- ☐ Algum episódio de disreflexia autonômica (dor de cabeça latejante, suor excessivo...) relacionado aos seus problemas intestinais
- ☐ Nenhum dos casos anteriores

Se o seu paciente apresentou um ou mais sintomas da lista acima, ele se qualifica para o status ⊕

Prossiga para o disco MENTOR para avaliar o tratamento com base neste questionário

Nome do paciente: ________________________

Data: ___________

Matriz de decisão do MENTOR

Passo 1 – Determine a faixa do NBDS

Ao somar os valores das 10 questões do NBDS, é obtida uma pontuação total que vai de 0 a 47. No eixo vertical da matriz, indique a faixa que corresponde à pontuação do paciente: 0-9, 10-13 ou acima de 14.

Passo 2 - Satisfação subjetiva do paciente

Uma vez identificada a faixa de NBDS que corresponde à pontuação numérica do paciente, identifique a escala de satisfação subjetiva do paciente mostrada por ele no eixo horizontal.

Passo 3 – Sintomas de atenção especial

Uma vez identificada a faixa de NBDS e o nível de satisfação do paciente, é necessário ver a seção do questionário referente aos sintomas que requerem atenção especial. Se o paciente não tem sintomas que requerem atenção especial, sua avaliação está terminada e a cor deve condizer com a descrição do resultado Verde (Monitorar), Amarelo (Discutir) ou Vermelho (Agir).

Se o paciente apresentar um ou mais sintomas de atenção especial, esse paciente deve mudar para um quadrado acima e um quadrado para a direita. Isso coloca o paciente para uma nova categoria de resultado e a avaliação do paciente agora deve condizer com a descrição do resultado Verde (Monitorar), Amarelo (Discutir) ou Vermelho (Agir).

Monitorar

Essa pontuação MENTOR sugere que o funcionamento intestinal do paciente está eficiente. Provavelmente não há necessidade de mudança no tratamento. Monitoramento da rotina é sugerido a menos que novos sintomas se desenvolvam.

Discutir

Essa pontuação MENTOR sugere que o funcionamento intestinal do paciente pode está ineficiente. Deve haver uma discussão, podendo ou não resultar em uma investigação mais aprofundada ou mudança no tratamento. Se o tratamento for modificado, deve ser considerado um monitoramento após 1-3 meses.

Agir

Essa pontuação MENTOR sugere que o funcionamento intestinal do paciente está ineficiente. Provavelmente, há necessidade de maior investigação e mudança no tratamento. Quando o tratamento for modificado, deve ser considerado um monitoramento após 1-3 meses.

ESCALA NBDS	14+				
	10-13				
	0-9				
		Bom	Adequado/ aceitável	Ruim	Muito ruim
